# A Study of Immigrant Latinas Perspectives of Caring for their Diabetes

**DOI:** 10.1007/s40615-022-01404-5

**Published:** 2022-09-06

**Authors:** Sharon K. Titus, Gina Quiles–Pollard

**Affiliations:** 1 School of Nursing, Azusa Pacific University, 701 E. Foothill Blvd, Azusa, CA 91702-7000 USA; 2 Tuscaloosa, USA; 3 Pruitt-Healty, Rome, GA USA

**Keywords:** Latina, Diabetes, Health, Adherence, Barriers, Immigrants

## Abstract

Hispanic–Americans are disproportionately affected by type 2 diabetes compared to non-Hispanic Whites. Five million adult Hispanic Americans are estimated to have been diagnosed with T2D. Among US Hispanics, Mexicans have the highest rate (14.4%) of diabetes. Further, Hispanics are also twice as likely as non-Hispanics Whites to die from diabetes, making it the fifth leading cause of their death and a serious health problem in Hispanic communities. Yet, little is understood of what rural immigrant Latinas do to care for their diabetes health. In-depth interviews (3 focus groups) and thematic analysis found 16 Latinas had T2D on average for 9 years; all emigrated to the USA from Mexico, lived in the USA for an average of 27 years, and worked (60%). Within the domain of “What do you do to take care of your health?” Latinas desired to adhere to exercise, controlled diet, and medications, but perceived a powerful barrier to a healthy life was the American lifestyle that included long work days, more money to purchase unhealthy foods and a desire for them, and a lack of time for other forms of exercise. Despite the Latina participants’ years of experience about living with T2D in the US, they still struggled to adhere to healthy behaviors. Future research should address the longer time Hispanic immigrants live in the US with the more at-risk they become for diminished health.

Hispanics are the fastest growing ethnic population in the United States (US). By 2060, the > 100 million are predicted to be nearly 28% of the US population [1]. Type 2 diabetes (T2D), a glucose intolerant disorder, has dramatically increased among Hispanics in the past decade; 5 million adult Hispanic Americans are estimated to have been diagnosed with T2D [2]. Among US Hispanics, Mexicans have the highest rate (14.4%) of diabetes [2]. Further, Hispanics are also twice as likely as non-Hispanics Whites to die from diabetes, making it the fifth leading cause of their death (seventh leading cause for entire US population) and a serious health problem in Hispanic communities [3].

Access-to-care is a well-known barrier for immigrants, but is even more challenging for certain populations such as Hispanics who have come to rural America to live and work [4, 5]. In Washington State, 13% (973,000) of the population are Hispanics, and about 32% of these are immigrants with most (23%) born in Mexico [6]. The socio-demographics of Hispanics living in Washington resemble other Hispanics throughout the nation: they have higher poverty levels, poorer health state, no or limited medical insurance, and the greatest need for access-to-care [7]. They also contribute greatly to the local economy. Further, 14% (330,000) of Hispanics who live in Washington have been diagnosed with T2D [8], yet limited data exists on the prevalence of T2D among Hispanics in rural communities [9]. Walla Walla county, located in rural Washington State, is ≥ 21.7% Hispanics [10]. Difficult to reach populations have unique health needs and ability to access care, yet very little is known about Walla Walla’s immigrant Hispanic population’s access-to-care needs, in particular among the Latina population. Diabetes is known to affect women differently [11], and women with diabetes may identify access-to-care issues differently [11], for example, increased heart attack risk, increased risk for eating disorders, and social impacts on diabetes management [12]. Taking care of their health is a challenge for people of all genders, but “can be even more challenging” for women [11].

This study was designed to better understand perspectives of access-to-care needs among Latinas with type 2 diabetes who live in rural Walla Walla, Washington.

## Methods

This study included critical social theory (CST), which asserts certain groups, such as immigrants, are considered vulnerable populations. CST can bring recognition of their own health situation through understanding and deconstruction of previously established ideologies and cultures that affect them [13]. CST helps participants in a social encounter understand their own and each other’s needs/wants to identify problems/solutions in a collective manner [14]. A community-based participatory research (CBPR) approach with community health partners, including Hispanic healthcare providers, guided the project [15]. Community participants were viewed as “holders of the knowledge” and included throughout the study [16]. For example, they recommended data collection occur using snowball sampling method in focus group settings. CBPR is commonly used to improve health outcomes among Hispanic population studies [17, 18].

Participants were recruited in 2016 to identify Latinas who lived in rural Walla Walla, Washington. Eligibility included adults who self–identified as having T2D, were immigrants to US, sought healthcare from any rural local health program, and consented to participate. Pregnant Latinas were excluded. Data collection occurred in English and led by a “moderating team” of a bilingual, immigrant Latina moderator and a co-moderator trained in conducting focus groups (*n* = 3) [19]. Participants chose their settings (2 local churches and 1 home). A semi-structured interview guide was developed with the tenets of CST and through feedback from the community health partners and from an expert in CBPR methods and health disparities research. The interview guide contained formal, predetermined questions, which helped guide the discussion and reduce the variation of the discussion [19]. Each focus group included a review of the activities and that their comments would be confidential. Participants were asked to focus their responses to questions as it related to having T2D and barriers to caring for their health.

Demographic data were analyzed descriptively using SPSS (IBM Corp Version 23). Conventional content analysis approach [20] was applied using triangulation of data through observation, field notes, and interviews. Audio recordings were translated verbatim and transcribed then independently double-checked for accuracy by the moderating team and one participant from each focus group. Field notes were collected from the early stages of the field work to the completion of the study and thematically analyzed to identify barriers to accessing care. Analysis occurred through a “back-and-forth” process until agreement was reached. Member checks occurred with Latinas from each focus group. Finally, a “Celebration Party” was held where Latinas from all focus groups collectively provided a consensus of the findings followed by a meal prepared by the participants. Each participant received a $25 gift card. The University of Hawaíi Institutional Review Board approved this study.

This paper reports on one question from the interview guide, “What do you do to take care of your health?” as it reflected the CST tenet of “engage client in critical recognition of their own health issues/situation” [21].

## Results

Each focus group lasted about 1 h. By design, all participants were immigrant Latina who came to the US on average 27 years ago, self-identified of Mexican descent (100%), and had been diagnosed with T2D on average of 9.31 years (Table 1).

**Table 1:** Participant demographics (*n* = 16)

Characteristic	Value labels			*N* (%)
Age (years)	18–30			1
	31–40			1
	41–50			8
	51–60			1
	> 60			5
Marital status	Married			13 (86.7)
	Divorced			1 (6.7)
	Single			1 (6.7)
Language spoken at home	Spanish			7 (43.8)
	Both Spanish and English			9 (56.3)
US citizen	Yes			6 (46.2)
	No			3 (23.1)
	Green Card			4 (30.8)
Work status	Full-time			4 (26.7)
	Part-time			5 (33.3)
	Not working/disabled			4 (26.7)
	Retired			2 (13.3)
Health insurance	Yes			9 (56.3)
	No			7 (43.8)
Type of health insurance	Medicare/Medicaid			6 (66.6)
	Private insurance			3 (33.3)
General health	Good			7 (43.8)
	Normal			3 (18.8)
	Poor			5 (31.3)
	Not sure			1 (6.3)
Do you go to a medical clinic?	Community clinic A			10 62.5)
	Community clinic B			3 (18.8)
	Other			3 (18.8)
*Additional questions of the participants*				
	*Range in years*			
	*Minimum*	*Maximum*	*Mean*	*SD*
How long have you been a diabetic?	1.0	32.0	9.31	9.98
When were you first diagnosed with diabetes by a doctor?	1.0	32.0	9.37	10.12
Number of years lived in the US	9.0	60.0	27.12	12.52

### Themes

**Fig. 1:**
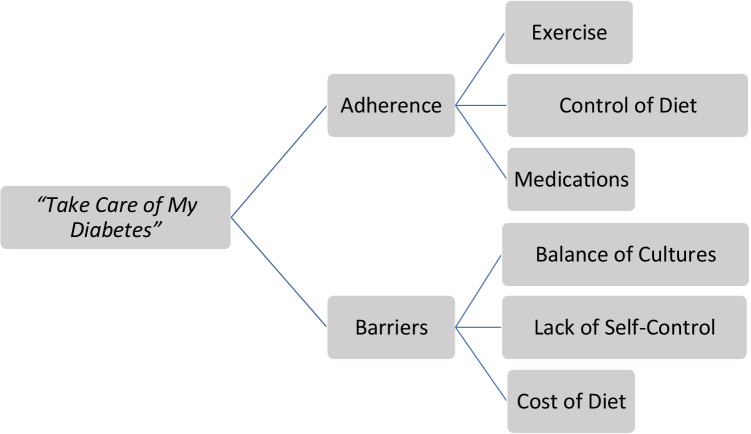
Qualitative Themes: “What do you do to take care of your health?”

Two themes and three subthemes for each were identified from the core interview question that focused on Latina’s overall experiences of taking care of their diabetic health (see Fig. 1). The following narratives support adherence factors to maintain health and barriers to accessing healthcare for immigrant Latinas with T2D. Table 2 illustrates the Latinas perspectives of caring for their diabetes health.

#### Theme 1: Adherence

The Latinas knew how to take care of their diabetes health, as most had been diagnosed years prior to the study. They all agreed on three particular activities to be healthy.

##### Exercise

The need to exercise was the most frequent response to adherence. Participants stated they gained exercise as they worked in the agricultural fields, at local canneries, one as a public health nurse, and some with busy household activities. Some explained how they exercised and the frequency of their exercise as shared by one participant, “Um, I mow lawns. I go walking. Actually, that’s why I was a little late, because I had to shower. Um, I don’t walk every day, but I should, but I do, I’m active. I––clean my house.” Other participants explained the consequences if they did not exercise. “If you don’t exercise it will contribute [T2D]. That’s what I think.” Another shared, “When we came to America, we worked long days at the pea cannery. But now we don’t work.”

##### Control of Diet

The participants identified a need to adhere to a controlled diet. Participants commonly stated there was an abundance of “green” vegetables grown in their rural agricultural community and that many had worked in the fields or canneries at one time. Several participants perceived that cooking at home was a healthier diet choice than eating on the streets. Eating on the streets was considered “fast foods.” Effective adherence was perceived as working with a local dietician, avoiding foods high in fat or carbohydrates, and eating more vegetables when at home. One participant said, “I know I needs to listen to what [dietician at clinic] is telling me, and uh, take heed to the advice that she has, and gets back on track [with her diet].” Some stated it was easier to control their diet if they ate at home. One stressed, “I now [try] to not to eat as much food in the street. Do more vegetables in the house.” In contrast, others felt it more challenging to adhere to their diet when they were home, “When I would stay home, I wasn’t working…I would eat [more].”

##### Medications

Participants identified that to properly care for their T2D meant, they needed to adhere to their daily medications. All participants reported that they had access to and utilized one of two local safety-net clinics for their medications with a few who had private insurance and used a pharmacy. They also reported that there were Spanish-speaking physicians and pharmacist at both safety-net clinics. One participant commented, “[Diabetes] to me is kind of the process of managing it with medication.” Another stated, “I need to stay on top of my medications.”

#### Theme 2: Barriers

The Latinas in all focus groups described three significant perceived barriers that kept them from adhering to activities that provided care for their diabetes health.

##### Balance of Cultures

All the participants had emigrated to the US from Mexico. Many fondly reminisced about a poorer life in their home country where they were financially impoverished and could not afford expensive foods or cars. Thus, they ate simple foods and commonly walked to the market place for groceries. Since immigrating to the US,“all we do is work, work, work” outside the home so much that “Latinas don’t pay attention to their own health.” Yet, several participants agreed that work in the U.S. gave them a higher income and access to and a desire for more expensive foods they believed were less nutritional and that contributed to their T2D. One participant spoke of how she had a marked changed in her diet as soon as she immigrated to America, “When you get into the U.S. that diet changes tremendously, where we eat a lotta junk! We really do, we eat a lot of sweet stuff and, because it’s all out there. You come from a poor family in Mexico, there’s no way you’re gonna go get pastries or donuts or whatever. You’re not gonna find that over there.” Another shared a similar comment, “Yes, [when I lived in Mexico] I ate chicken soup, with greens…but now I eat hamburgers, pizza.” Finally, one participant specially shared what healthy meals cooked at home in Mexico included. “Uh, beans, rice, tortillas, salsa, and meat…and then legumes. Lentils. I mean, that is an excellent diet…home-cooked meals [are more nutritious]! (Tables 2 and 3).

**Table 2:** Illustrative quotes for “Taking care of my diabetes”

Themes and subthemes	Illustrative quote
*Theme I: Adherence*	
Exercise	“Um, I mow lawns. I go walking. Actually, that’s why I was a little late, because I had to shower. Um, I don’t walk every day, but I should, but I do, I’m active. I––clean my house” (FG 1) “If you don't exercise it will contribute [T2D]. That’s what I think” (FG3) “When we came to America, we worked long days at the pea cannery. But now we don’t work”(FG1) “We eat together, why shouldn’t we exercise together” (FG2)
Control of diet	“I know I needs to listen to what [dietician at clinic] is telling me, and uh, take heed to the advice that she has, and gets back on track [with her diet]” (FG 2) “I now [try] to not to eat as much food in the street. Do more vegetables in the house” (FG 2) “When I would stay home, I wasn’t working…I would eat [more]” (FG 3)
Medications	“[Diabetes] to me is kind of the process of managing it with medication” (FG 2) “I need to stay on top of my medications” (FG 2) “My sugars are normal with the medications (FG 1)
*Theme II: Barriers*	
Balance of cultures	“All we do is work, work, work” (FG 2) “I think many Latinas don’t pay attention to their health” [because we work] (FG 3) “When you get into the United States that diet changes tremendously, where we eat a lotta junk! We really do, we eat a lot of sweet stuff and, because it’s all out there. You come from a poor family in Mexico, there’s no way you’re gonna go get pastries or donuts or whatever. You’re not gonna find that over there” (FG 2) “Yes, [when I lived in Mexico] I ate chicken soup, with greens…but now I eat hamburgers, pizza” (FG 1) “Uh, beans, rice, tortillas, salsa, and meat…and then legumes. Lentils. I mean, that is an excellent diet…home-cooked meals [are more nutritious]! (FG 1)
Lack of self-control	“It’s like if I’m gonna die, I’m gonna die happy, drinking what I like! (FG 3) “I struggle because I really like bread, sodas, tortillas and all the things that have fat (FG 3) “[to follow a healthy diet] Exactly see, that’s the kind, for me that’s a barrier, big times! (FG 1)
Cost of diet	“My family, hasn’t diabetes. My grandparents, my, nobody in…my mother passed away last month [They live in Mexico where they are poor and can’t afford to eat junk food so Mexicans in Mexico do not have T2D]” (FG 1) “The other barrier: things that are healthy are more expensive…You can get two Whoppers for five dollars…by the time you buy the meat, vegetables, you spend more than that” (FG 1)

*FG* focus group

**Table 3:** Interview guide

Critical social theory core tenet	Dimension	Core question	Follow up questions and prompts
Assess how things are in order to transform them into what they ought to be [22]	Normative cultural values of Latinos about the concept of health	What does health mean to you?	Do you think some Latinas might view health differently than you? What do you think they may say?
Engage client in a critical assessment of health issues/situation that affect Latina care [23]	Significant health issues that need to be followed up	What do you think are the most important health issues faced by Latinas today?	What about diabetes? How important do you think diabetes is? Is it a very important issue, one of the most important, a somewhat important issue, or not too important of an issue? Can you tell me why you rated it this way?
*Engage client in critical recognition of their own health issues/situation* [21]	*Health maintenance*	*What do you do to take care of your health?*	*Can you give me some examples of things you do [to take care of your health]?*
Engage client to transform and improve health care access and practices in managing and controlling T2D [14]	Health maintenance	How can you tell you are having trouble with your health?	So what do you do when you’re having trouble with your health [don’t feel well]? Are there specific people or places you go to get help with your health? How do you figure out where to go to get help?
Engage clients to illuminate health care structures that may compromise their care [13]	Location of health services	Where do you go to get care for your health? (type of service or name of clinic)	(for each one mentioned) What are your reasons to choose to go there?
Engage clients to illuminate health care providers who help them value their autonomy and responsibility [24]	Person(s) go to for health issues	Who do you go to get care for your health issues?	What are your reasons to choose to see this person(s)?

##### Lack of Self-Control

Many participants described their desire to eat a healthy diet, but some perceived that lack of self-control was a main barrier. One stated, “It’s like if I’m gonna die, I’m gonna die happy, drinking what I like!” Another added “I struggle because I really like bread, sodas, tortillas and all the things that have fat.” And one stated, “[to follow a healthy diet] Exactly see, that’s the kind, for me that’s a barrier, big times!”.

##### Cost of Diet

Participants said they could control their diet if they could afford healthy foods. They stated healthy foods in Mexico, such as fruits and vegetables, were less expensive to eat. They found food in America was expensive, and suggested that people “back home” do not even get diabetes because they cannot afford to purchase bad foods, “My family, hasn’t diabetes. My grandparents, my, nobody in…my mother passed away last month [They live in Mexico where they are poor and can’t afford to eat junk food so Mexicans in Mexico do not have T2D].”

## Discussion

This study illuminates immigrant Latinas perspectives of the access to activities needed to live a healthy life with diabetes in a rural agricultural county of America. Participant voices of adherence activities and barriers to adherence reflected their previous “life in Mexico” with their current life in rural America.

A powerful finding in our study was their recurrent discussion of “life in Mexico” where the participants’ perceived people do not have the prevalence or severity of diabetes than those who have immigrated to the US. They specifically spoke about traditional lifestyles to exercise and diet in Mexico, which helped them to live a healthy life. The Latinas reminisced about their life in Mexico where people are poor, and, therefore, they exercised daily with activities that are free, such as walking to the market place to buy meat, rice, and legumes. Because they were poor, some laughed that they could not even afford “sweets” or “junk food.” They also shared that in Mexico, women traditionally made “home-cooked meals.” Being poor, the need to walk to purchase food, and meals at home led to an “excellent diet” and overall good health. However, after migration, Latinas reported life “changed tremendously” as they now worked a lot and could afford a more expensive lifestyle, like cars to drive for shopping and fast-foods. The Latinas expressed the America lifestyle of work and diet contributed to their diabetes health. Other studies support that immigrant Hispanics have acquired poor exercise [25] and dietary habits [26] that have contributed to their diabetes and that some may have changed their lifestyle in a desire to “fit in” after immigrating to the US [27] even though they believed it increased their likelihood of developing T2D [28]. Additional studies are warranted to explore the “immigrant paradox” experienced among Hispanic women whose health are impacted by factors such as change in their socioeconomic status and a “balance of culture” after settling into their new country [29].

Another important finding was the Latina responses related to their rural agricultural community. Most immigrated to Washington State to work in the fields or vegetable canneries where they reported getting a lot of “work, work, work” related exercise that helped them to live healthy. In contrast, others stated they worked so much that they did not “pay attention” to their own health. Years later, most transitioned from working in the fields or canneries to other work environments that were less physically demanding. Some shared they no longer worked “long days” but continued “to try” to exercise, as there were consequences if they did not exercise, such as their diabetes would worsen. Previous studies indicate that 40% of Hispanic women with T2D (Mexican 71%) never exercised, only 20% exercised once or twice per week [30], and that nationally, Hispanics are less likely to exercise than non-Hispanics whites [31]. Hispanic immigrants (78% from Mexico) have been demonstrated to believe “exercise is a battle” in managing their T2D even when they had an exercise equipment [32]. Important to rural health is access to community exercise programs to help Hispanics adhere to daily exercise habits [33]. To decrease barriers to exercise activity and improve daily exercise habits, there is a need for access to public recreation programs, as well as culturally appropriate [34] exercise resources for Hispanics who live in rural communities [35]. For example, fitness equipment in public parks may increase access to affordable physical activity [36].

Participants in the current study desired to adhere to a controlled diabetic diet, and they spoke of their need to eat more “green foods,” control their portion sizes, and adhere to advice by the health clinic or health fair dietician. Additionally, because they lived in a large agricultural community, they stated they had access to plenty of nutritious foods. Yet, several recognized the high cost of some nutritious foods that were suggested to them by the local dietician and that they also liked to eat fast-foods. Similar to their exercise habits, many participants prefaced their thoughts with the words such as “I need to get back on track” or spoke of lacking self-control, suggesting other factors may also influence their ability to adhere to a controlled diet.

Finally, our study found participants believed medication adherence was needed to live a healthy life with diabetes as the Latina reported how many medications they took and where they got their medications. No participants reported any barrier to medication adherence. This suggests the unique immigrant Latina population in this study as the participants had had diabetes for many years, had some form of health care insurance, and had routine access to health clinics in their community spaces that provided low-cost medications and were staffed with pharmacy providers who spoke Spanish.

In summary, the participants voiced that adherence to exercise, diet, and medication were needed to maintain their diabetes health. However, a powerful barrier to a healthy life was the American lifestyle that included long work days, more money to purchase unhealthy foods and a desire for them, and a lack of time for other forms of exercise. Future research must continue to include models that help Latinas overcome barriers to health care access in rural Latino communities [37].

## Limitations

Limitations including the snowball recruitment sampling may have limited the findings to a select rural Latina population. Although all participants spoke English, their level of English fluency was not known and may have contributed to their level of participation in the focus group discussions. This was evident during a few times, when a participant struggled to say something, the Spanish speaking moderator would ask the participant to respond in Spanish. Future research should recruit Latinas who have resided in the US longer and shorter times to study whether the length of time they reside in the US impacts their adherence to living a healthy life and to determine if unmarried Latina’s barriers are unique compared to the married sample (87%) found in this study.

## New Contribution to the Literature

This study provides qualitative evidence that, although the Latina participants had years of experience and knowledge about living with T2D, they still “struggled” to adhere to exercise and diet behaviors due to barriers they perceived were related to cultural factors, including a previous “life in Mexico” and the demands of the American lifestyle of “work, work, work!” The interview guide, based in critical social theory, was paramount for the immigrant Latina to critically share what they wanted the moderating team “to know” about their diabetes health as participants enthusiastically shared, “Oh! You mean we get to tell you [about what we feel]? Always we go to places, and they tell us. The clinic tells us. The dietician tells us. The health fair tells us. It’s the reverse here! This is the first time we get to tell them! (focus group 3).
